# Studying metabolism with multi-organ chips: new tools for disease modelling, pharmacokinetics and pharmacodynamics

**DOI:** 10.1098/rsob.210333

**Published:** 2022-03-02

**Authors:** Tanvi Shroff, Kehinde Aina, Christian Maass, Madalena Cipriano, Joeri Lambrecht, Frank Tacke, Alexander Mosig, Peter Loskill

**Affiliations:** ^1^ NMI Natural and Medical Sciences Institute at the University of Tübingen, Reutlingen, Germany; ^2^ Department for Microphysiological Systems, Institute for Biomedical Engineering, Faculty of Medicine, Eberhard Karls University Tübingen, Österbergstraße 3, 72074 Tübingen, Germany; ^3^ Institute of Biochemistry II, Center for Sepsis Control and Care, Jena University Hospital, Jena, Germany; ^4^ esqLABS GmbH, Saterland, Germany; ^5^ Department of Hepatology and Gastroenterology, Charité University Medicine Berlin, Campus Virchow Klinikum and Campus Charité Mitte, Berlin, Germany; ^6^ 3R-Center for In vitro Models and Alternatives to Animal Testing, Eberhard Karls University Tübingen, Tübingen, Germany

**Keywords:** multi-organchip, *in vitro* to *in vivo* translation, *in silico* modelling, metabolism, PK/PD, disease modelling

## Abstract

Non-clinical models to study metabolism including animal models and cell assays are often limited in terms of species translatability and predictability of human biology. This field urgently requires a push towards more physiologically accurate recapitulations of drug interactions and disease progression in the body. Organ-on-chip systems, specifically multi-organ chips (MOCs), are an emerging technology that is well suited to providing a species-specific platform to study the various types of metabolism (glucose, lipid, protein and drug) by recreating organ-level function. This review provides a resource for scientists aiming to study human metabolism by providing an overview of MOCs recapitulating aspects of metabolism, by addressing the technical aspects of MOC development and by providing guidelines for correlation with *in silico* models. The current state and challenges are presented for two application areas: (i) disease modelling and (ii) pharmacokinetics/pharmacodynamics. Additionally, the guidelines to integrate the MOC data into *in silico* models could strengthen the predictive power of the technology. Finally, the translational aspects of metabolizing MOCs are addressed, including adoption for personalized medicine and prospects for the clinic. Predictive MOCs could enable a significantly reduced dependence on animal models and open doors towards economical non-clinical testing and understanding of disease mechanisms.

## Graphical abstract

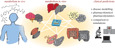

## 1. Introduction

Metabolism consists of three categories of life-supporting functions [1]. The first involves the biochemical events across tissues that generate or consume energy to maintain homeostasis. The second involves the production of building blocks such as proteins and lipids from food, to support body functions. The final aspect of metabolism involves the elimination of metabolic waste and xenobiotics. The primary organs involved in metabolism include the gut, liver, adipose tissue, pancreas, kidney and muscles. During metabolic regulation, these organs interact through different signalling pathways elicited by hormones and morphogens, providing the body with the appropriate amount of energy it needs. Immune-related processes also affect metabolic function through multiple mechanisms [2].

Metabolism is regulated by complex signalling pathways that centrally involve hormones such as insulin and glucagon secreted by the pancreas, provoking effects in energy-consuming organs including the muscle, liver and adipose tissue. The secretion of these signalling molecules differs with glucose availability, which regulates the adaptive metabolic response. Dysfunction of metabolism has been linked to the onset of a variety of diseases caused by disturbance of homeostatic mechanisms required to maintain proper cell function.

### 1.1. Types of metabolism

Carbohydrate metabolism is closely regulated by insulin and glucagon, both of which are secreted by the pancreas. When glucose-containing meals are ingested, the pancreas secretes insulin, leading to elevated glucose uptake by the muscles and liver; under fasting conditions, the pancreas secretes glucagon, promoting glucose metabolism from stored glycogen. This regulation forms the basis of glucose metabolism. Stored lactate is a hydroxycarboxylic acid produced from glucose by glycolysis or the pentose phosphate pathway or by the transamination of alanine. Monocarboxylate transporter 4 is responsible for the secretion of lactate from cells. While exercising or during a state of starvation, lactate becomes a precursor for gluconeogenesis [3]. Lipid metabolism involves the uptake and release of free fatty acids (FFAs) into the circulation, mediated by pathways involved in signalling with adipose tissue. Adipokines, secreted by adipose tissue, have been implicated in the progression of insulin resistance during obesity. Lipids make up many key cell components but the dysregulation of lipid metabolic pathways leads to intracellular lipid deposition and functional dysregulation of cells [4]. Protein metabolism is influenced by various factors such as food, hormones, inflammatory stimuli and age. Some hormones have catabolic effects (glucagon, glucocorticoids and catecholamines), others have anabolic effects (insulin, insulin-like growth factor 1 and growth hormone) and the effects of some are unknown [5]. Essential amino acids are consumed in the diet and subsequently acted upon by branched chain amino acid transferase, after which they undergo a series of transformations to finally become products that get rerouted to the tricarboxylic acid (TCA) cycle, like succinyl CoA, etc. Valine is glucogenic; isoleucine is glucogenic and ketogenic while leucine is ketogenic. Protein synthesis and protein breakdown determine the homeostasis of proteins in the body [6]. Drug biotransformation refers to the process by which xenobiotics are enzymatically converted to make them readily excretable and to eliminate pharmacological activity [7]. This mainly involves changes to the drug iso-form or addition of functional groups, making the parent drug molecule more hydrophilic and prone to elimination. The liver is one of the key organs where drug metabolization occurs, closely followed by the intestine and kidneys and other organs to a lesser degree (e.g. heart, blood, skin and brain). Drug-metabolizing enzymes can be classified into two phases (phase 1 and phase 2) depending on the type of metabolism they carry out.

### 1.2. Physiology of inter-organ communication and the disruption of metabolic pathways

Metabolism involves harmonious signalling between multiple organs and, depending upon the type of metabolism to be studied, it is imperative to understand the physiology of organ interactions. Human metabolism involves multiple organs and specialized tissues to digest, store and retrieve energy from nutrition. One of the central metabolizing organs is the liver. The broad range of different biotransformation processes is mainly executed by hepatocytes. Liver parenchymal cells (i.e. hepatocytes and cholangiocytes) are surrounded by a network of non-parenchymal cells (e.g. liver macrophages (Kupffer cells), sinusoidal endothelial cells and hepatic stellate cells) in the hepatic sinusoid that contribute to and regulate metabolic activities and immunological responses. Depending on their localization in periportal, mid- and pericentral zones of the hepatic lobules, hepatocytes are exposed to various biophysical and biochemical cues. The liver and the adipose tissue are specialized organs for storing energy. Adipose tissue makes up 20–50% of body weight and functions as a storage organ for fatty acids in addition to being a powerful endocrine organ. There is evidence that the visceral fat influences metabolism via the secretion of adipokines and FFAs [8]. Skeletal muscle tissue represents the most abundant muscle type in the human body and constitutes about 40–45% of total body weight, but 70–80% of cell mass. Besides providing mechanical forces for movement, muscle tissue also serves as an energy depot. It is a vital storage site for proteins and free amino acids, which represent a major source for generating glucose through gluconeogenesis to ensure a glucose supply for the brain during starvation. The pancreas is a compound gland that discharges digestive enzymes into the gut and secretes the hormones insulin and glucagon, which play important roles in glucose metabolism, into the bloodstream. These two hormones influence the rate of glucose breakdown in the body [9]. The small intestine covers a considerable length and is one of the first sites of absorption of nutrients and xenobiotics into the bloodstream [10]. The intestinal epithelial cells have a large surface area for absorption and metabolism, and the ability to regenerate, thus maintaining intestinal function. The presence of immune cells and the gut microbiota contributes significantly to the intestine's metabolizing capabilities [11].

The various metabolizing organs in our body interact synchronously to maintain homeostasis. The liver and adipose tissue are the vital metabolic organs necessary for energy utilization and storage. Both organs interact for the regulation of the metabolism of lipid and glucose by secreting various growth factors with a vast range of metabolic regulatory effects, including fibroblast growth factor 21, adiponectin (APN) and the pro-inflammatory factors adipose fatty acid-binding protein-4 and lipocalin-2 [12]. The pancreatic islets of Langerhans are endocrine structures distributed throughout the exocrine portion of the pancreas. They consist of five distinct cell types: α cells (secreting glucagon), β cells (insulin), γ/PP cells (pancreatic polypeptide), δ cells (somatostatin) and ε cells (ghrelin). In the liver, insulin plays a major role in glycogen synthesis and inhibits glycogen breakdown. Additionally, it regulates glycolysis and inhibits gluconeogenesis, which influences glucose metabolism. Insulin also performs different anabolic functions in the liver, stimulating lipid synthesis and release and protein synthesis and inhibiting the breakdown of these substances [13]. Skeletal muscles secrete myokines and peptides that can perform autocrine, paracrine and endocrine actions. Exercise contributes to their release and is shown to produce adipocyte browning. Myonectin is involved in fatty acid uptake and oxidation in adipose tissue and liver. Insulin resistance in the skeletal muscle changes the expression of these myokines and influences fatty acid metabolism in the body [14]. Myokines counteract the effects of adipokines. Myostatin is one such myokine and is important for the maintenance of metabolic homeostasis and in regulating the size and function of adipose tissue. Interleukin 6 (IL-6) is secreted into the bloodstream in response to muscle contractions during exercise, not because of immune responses in this state [15]. IL-6 is also hypothesized to increase fatty acid oxidation through AMP-activated protein kinase and is responsible for glucose production during exercise but might worsen insulin resistance between liver and adipose tissue.

Adipose tissue has revealed itself as a major endocrine regulator, impacting the metabolic balance in the body and hence leading to numerous pathophysiologies downstream. The link between obesity and the metabolic syndrome is critically dependent on the distribution of body fat. Clinical data show that abdominal obesity is more strongly associated with the development of the metabolic syndrome than peripheral body fat distribution, proving the critical role of visceral fat in the development of metabolic diseases. Obese adipose tissue secretes adipokines, thereby causing damage to pancreatic β cells [16]. Thus, the cross-talk between pancreatic adipocytes and islets is defined by the metabolic status, which in turn regulates the secretion of adipocytes and islet activity and, therefore, the paracrine effects. Adipocytes secrete leptin and APN. Leptin is a peptide that is produced by mature adipocytes and that acts mainly on the central nervous system. Leptin release is regulated by cross-communication of fat tissue, brain and bone and acts on the β cells of the pancreas by negatively regulating pancreatic β cell function and cell mass [17,18]. However, some of the endocrine actions of leptin on β cells are not mediated via its receptors on β cells. Leptin regulates bone metabolism, which demonstrates that bone may exert feedback control on β-cell function [19]. APN target organs include the liver, where the hormone counteracts gluconeogenesis; the skeletal muscle, where it stimulates oxidation of fatty acids; and the brain, where it ensures a continuous energy supply [20]. Under diseased conditions, adipokines, pro-inflammatory cytokines, FFAs and other substances are released by adipose tissue, thereby contributing to a hepatic acute-phase response. The secreted pro-inflammatory cytokines stimulate liver resident macrophages (Kupffer cells), which mediate inflammation that interferes with the secretion of regulatory factors of lipid and glucose metabolism. This pathophysiological effect is thought to be causative for the observed association between dysregulation in lipid and glucose metabolism and deregulation of metabolic signalling pathways in gluco-metabolic diseases [21].

Immuno-metabolism represents a key mechanism central to innate and adaptive immune regulations. Early studies identified inflammatory cytokines secreted in obese adipose tissue as drivers of metabolic disease by initiating the cross-talk between immune cells and metabolism, resulting in aggravation of the inflammatory loop [22,23]. Metabolic pathways are intricately linked to cell signalling and differentiation, giving rise to different immune cell subsets that adapt in response to biochemical and biophysical cues of their micro-environments, inducing unique metabolic fates.

### 1.3. Non-clinical models to study metabolism

Metabolism has been intensively studied in various *in vitro* models, including precision-cut tissue slices as well as cell lines and primary cells that could be genetically engineered for the expression of specific enzymes [24]. Advanced *in vitro* systems such as three-dimensional cultures and organ-on-chip (OoC) systems could present advantages for metabolism-based studies, in being able to isolate certain pathways and study disease pathophysiology. Organs-on-chips recreating specific organotypic functions can enable the understanding of the development of a certain disease pathophysiology through the lens of one specific target organ for a specific donor [25]. Furthermore, they can be specifically tailored to study a specific organ function by integrating non-parenchymal cells that improve the physiological relevance of the organ model [26]. On one hand, single-organ chips could serve as a framework to identify vital biological mechanisms such as disease progression and immune–organ interactions to identify key biological pathways and new targets for drug testing. This leads to the next application of organ chips to test drug efficacy and metabolism in target organs at the pre-clinical development stage, thus providing a reliable data source for clinical trials [27].

To study the full range of inter-organ interactions and identify potential toxicity of drugs and metabolites, two-dimensional cultures and animal models are widely used in biomedical research and drug development. However, these models are limited in physiological relevance and often differ significantly in (drug) metabolism from humans. Systemic studies have so far mostly been performed by use of *in vivo* models, i.e. rodent models, which show considerable interspecies variability in metabolic specificity, hormone regulation and thermal biology [28,29]. Two-dimensional models lack the complexity of the three-dimensional tissue microenvironment and the important cross-talk between different cell types and the extracellular matrix. Studies involving animals are often challenging to extrapolate to humans as different animal species give conflicting results, e.g. on drug toxicity. *In vitro* models that are able to recapitulate the cross-talk between metabolic tissues would be a major step forward for metabolism-associated research. Especially to model metabolic diseases, complex bioengineered three-dimensional model systems that are physiologically representative of the tissue microenvironment are urgently required [30]. Although the systemic clinical manifestation of metabolic disease cannot be fully recapitulated in a single tissue or organ model, understanding the basic processes can still show useful information about the whole disease mechanism. To model the spatio-temporal dynamic processes in the human body and disease pathogenesis, integration of these three-dimensional models by linking multiple organs together with functional vasculature will be crucial.

OoC systems belong to a new wave of *in vitro* models, which have the potential to better recapitulate metabolic physiology *in vitro* by allowing reliable control of cellular, biochemical and biophysical cues in a precise and accurate manner [31,32]. This highly interdisciplinary technology combines microfabrication techniques, tissue engineering and (stem) cell biology to create perfused species-specific *in vitro* models to answer specific scientific questions related to disease mechanisms or drug response [33]. The underlying technological concept of microfluidics paves the way for the linkage of individual organ models by connecting perfused microchannels. Further applying mathematical modelling principles to the data from multi-organ chips (MOCs) will enable the generation of predictive *in silico* models that could predict human-scale metabolic responses.

**Figure 1.**
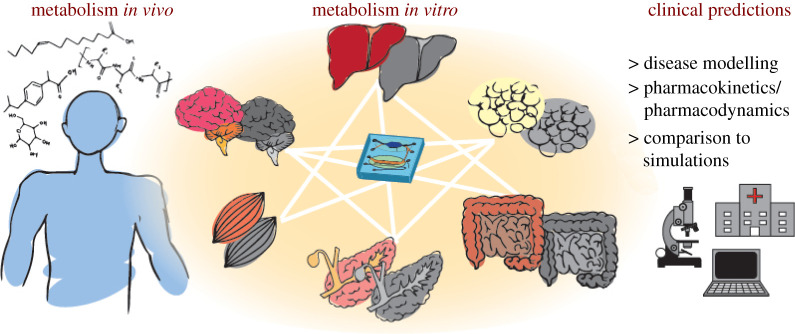
Schematic overview of the focus of this review—the study of metabolism spans many organs via various pathways. MOCs coupled with *in silico* models provide a strong platform in the prediction of disease progression, PK and PD.

In this review, we comprehensively review MOC models relevant for metabolism research towards clinically relevant predictions of disease progression, absorption, distribution, metabolism, excretion and toxicity (ADMET) and pharmacokinetics and pharmacodynamics (PK-PD; figure 1). We introduce the technical aspects of MOC design and strategies to correlate MOC data to predict *in vivo* outcomes, as well as further discuss specific application of OoCs in disease modelling, applications for mechanistic studies in ADMET and quantitative PK-PD modelling.

## 2. Concepts for linking multi-organ chips

Single-organ chips enable the study of organ-specific questions [34–36]. These include organ-specific PK aspects such as absorption in the gut, partitioning of the drug into adipose tissue, liver metabolism of the drug or excretion of the drug in the kidney. They also enable the study of disease mechanisms triggered in different organs, such as inflammatory bowel disease, asthma or liver disease [36–38]. Complex (patho)physiological processes can be captured by models integrating organ-specific extracellular matrix, multiple cell types or aspects of the immune system. The interconnection of OoCs to MOCs presents the opportunity to investigate organ cross-talk and address questions in a highly controlled manner, on a systemic level that can be compared with animal experimentation. Individual OoCs have metabolic attributes and can interact with each other and exchange substances via microchannel systems. Compared with the isolated culture of individual organ models, the organotypic functions in MOCs could be improved by their close interaction with other organs. To link multiple organ models, complex connection strategies are required. This section provides an overview of connection approaches. For more specific reviews of technical design, the reader is referred to additional literature focusing on designing organ chips, connection strategies for multiple organ models and approaches for appropriate organ scaling [39–41].

A physiologically relevant connection strategy of OoCs involves organ communication via simulated vasculature structures. This can be achieved by enabling flow of medium from one tissue chamber to another via a common fluidic channel. A monolayer of endothelial cells lining the connection channel could further allow for physiological exchange of signalling molecules across the endothelial barrier. Connections can be built into one single chip, where compartments for various tissues with a fixed cell ratio are perfused with a common cell medium flowing through fluidic channels built directly into the chip [42]. Since each tissue requires a different microenvironment, interconnected OoCs can be limited in terms of design flexibility [43,44]. Modular connections enable the culture of organ chips separately until the tissue is fully formed. Tissue models can be linked in a modular and flexible fashion. This type of system has several detachable parts and, hence, a higher likelihood of leaks, but provides a high degree of flexibility, allowing, for example, the insertion of sensors into the modular connection. Some examples of this methodology include Lego-inspired capillary connectors [45,46], insert-based connectors [47], three-dimensional bioreactors connected via tubing [48] or magnetic snap-fit connection systems [49]. In the case of fluidic interfaces, multiple chips can be plugged into a common interface with one inlet and outlet, while the chips are connected to each other via the fluidics built into the interface. The MOC designer is, however, restricted to the dimensions of the fluidic interface in terms of locating ports to connect the chip to the interface. This system makes it easier for automation and robotic handling owing to the fixed spatial arrangement of sampling ports, as demonstrated by Novak *et al.* [50].

A variety of perfusion approaches exist to flow media through MoCs. Passive flow represents an approach to using gravitational hydrostatic pressure difference, or surface tension to guide flow through channels [41]. This perfusion approach allows direct access to reservoirs at the inlet and outlet for medium change or effluent collection. The passive flow is usually created by rocking the devices back and forth [51]. Syringe or peristaltic pumps as well as centrifugal or pneumatic systems are able to create active, mechanically driven perfusion of medium. Syringe and peristaltic systems require the use of tubing, leading to a larger dead volume than passive flow and a higher tendency of leakage and bubble formation [52]. Syringe pumping is commonly used to circulate fresh medium in a unidirectional manner, allowing for the study of downstream effects of individual OoCs. Peristaltic pumping allows the recirculation of medium or mixing of recirculated and fresh media in a controlled ratio [53]. Centrifugal force represents a novel, innovative method that uses a spinning mechanism to perfuse medium and allows for the control of flow rates by regulation of the rotation speed [54]. Pneumatic pressure flow does not require the use of tubing and thus reduces the amount of required medium volume. However, these systems rely on gas supply channels to drive medium flow by membranes and need to be airtight in order to prevent pressure imbalance causing unpredicted detrimental flow conditions [55]. Electromagnetic actuators enable automated flow control for unidirectional or recirculating flow [56,57]. A more detailed overview of pumping approaches for an OoC platform is provided in a recent review by Byun *et al.* [58].

During the prototyping phase, the choice of material of construction of MOCs depends on the scientific application of the organ chips and scale of operation. Polydimethylsiloxane (PDMS) is gas permeable and ensures that the medium is saturated with gases at the same rate as the environment around it. Rigid plastics such as polymethyl methacrylate (PMMA) or cyclic olefin copolymer (COC) allow for locally tunable gas concentrations around the tissue depending upon the requirements of the study (e.g. anaerobic microbes in the gut or oxygen-dependent zonation in the liver) [59,60]. The absorption and adsorption of small molecules into the material of construction of the MOC must be monitored in order to ensure adequate dose exposures to the tissues for PK and PD studies [61].

An optimized combination medium is essential to ensure the proper functional state of each tissue in the MOC. This was demonstrated in a four-organ system by Oleaga *et al.* [62], who connected functional cardiac, neuronal, muscle and liver modules over an extended time period of 14 days via vascular perfusion of a common medium. Liver function was confirmed via albumin and urea production, cardiac and skeletal microtissue function was assessed in their contractile response to broad-field electrical stimulation and neuronal function was analysed using patch clamp electrophysiology. The integrated modules were exposed to drugs with known side effects for 7 days to show correlation with published human and animal data. The authors also attempted to investigate animal serum-free medium to ensure better control over MOC culture conditions [62]. In a similar approach, Miller & Shuler [43] presented a 14-organ MOC which remained functional over a 7-day period and was perfused via gravity-driven flow. While these systems lacked an endothelial barrier layer across which nutrient exchange could take place, they provide insights into functional MOCs with common circulating media.

To monitor metabolic activity in MOCs, the effluent or perfused medium is typically analysed outside the chip. A number of approaches, however, integrate in-line sensors, which can be used for *in situ* readout of metabolic function [63]. An interesting development is the integration of enzyme-based multi-analyte biosensors into a multi-tissue culture platform for MOC application. Misun *et al*. [64] developed a multi-tissue platform with integrated sensors for lactate metabolism. The sensor modules were designed as small glass plug-ins featuring platinum working electrodes, coupled with oxidase enzymes to allow continuous measurement of lactate and glucose. The biosensors recorded high sensitivities of 443 ± 37 nA mM^–^^1^ mm^–^^2^ for lactate; the corresponding limits of detection were below 10 μMT. The model enabled tissue-size-dependent, real-time measurement of lactate secretion from the three-dimensional microtissues cultured in a hanging drop configuration [64]. Glieberman *et al.* [65] demonstrated a high-throughput pancreatic islet capture chip with in-line insulin sensing during glucose-stimulated insulin secretion experiments. Taken together, the use of integrated sensors for glucose, ammonia, insulin, oxygen [66] and reactive oxygen species [67], among others, will allow for in-line data acquisition that can support *in silico* modelling. That being said, in-line sensing is still an important challenge for the field, owing to the limited number of analytes, sensor robustness and fabrication limitations.

## 3. Multi-organ chips to study metabolism

**Figure 2.**
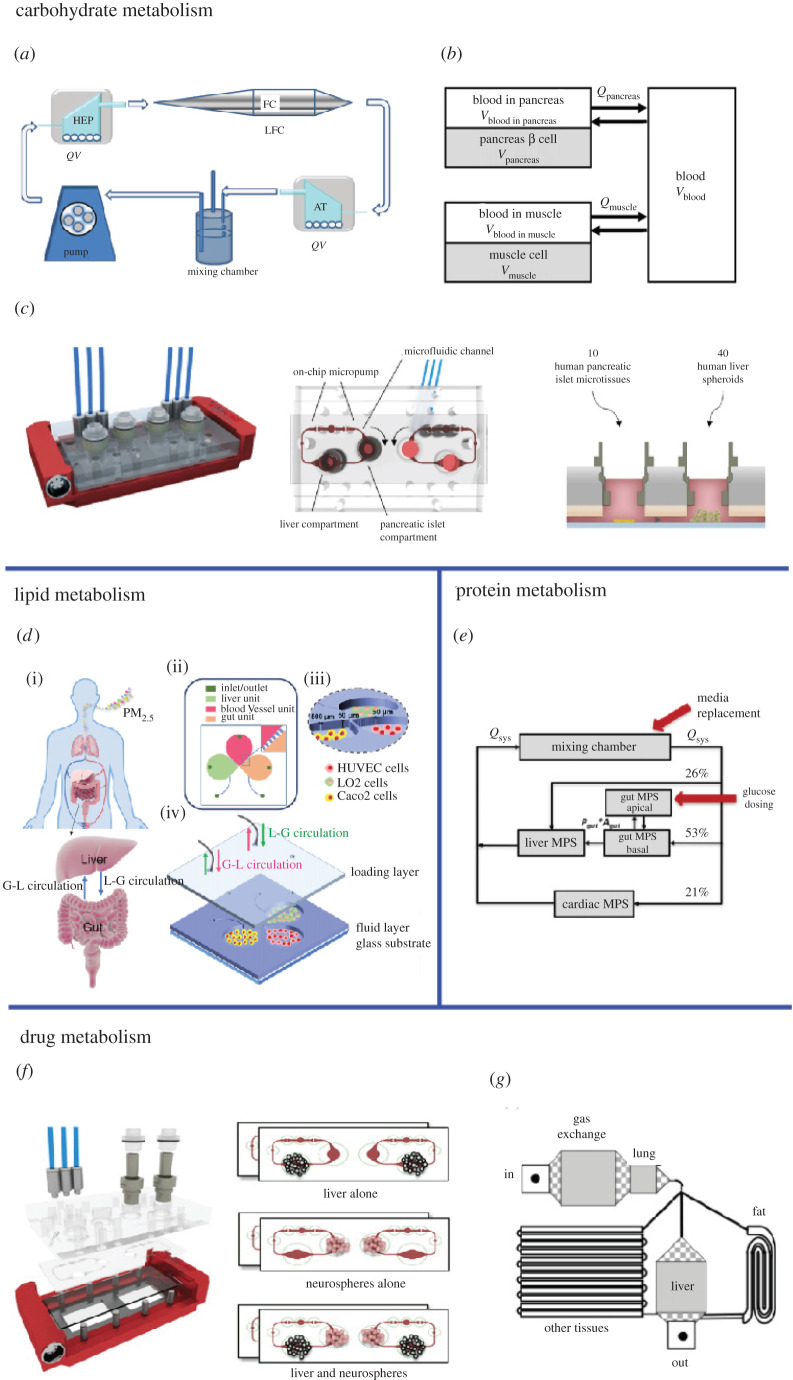
MOCs to study the various types of metabolism. Carbohydrate metabolism—(*a*) a three-way connected organ system featuring adipose, liver and vascular tissue (adapted from [68] (CC BY 4.0)), (*b*) a connected pancreas–muscle tissue model to study insulin-dependent glucose uptake (adapted from [69] © 2019 Wiley Periodicals, Inc.) and (*c*) a model connecting pancreatic islets and liver spheroids to study insulin signalling between the cell types (reproduced from [70] (CC BY 4.0)). Lipid metabolism—(*d*) the study of cholesterol dysregulation using a gut–liver-on-chip model (adapted with permission from [71]. Copyright 2021 American Chemical Society). Protein metabolism—(*e*) connected gut–liver–cardiac system for the study of protein metabolism (adapted from [72] (CC BY 4.0)). Drug metabolism—(*f*) a neurotoxicity study involving the interaction between liver and neurospheres (reprinted from [73] © 2015 with permission from Elsevier) and (*g*) accumulation, distribution and toxicity in a liver–fat–lung-on-chip system (adapted from [74] (CC BY 4.0)).

This section provides an overview of the literature involving MOCs that study the four previously described types of metabolism—carbohydrate, lipid, protein and drug metabolism (figure 2).

Lee *et al.* [69] designed a three-organ chip system of pancreas, muscle and liver models to mimic glucose metabolism and homeostasis. An *in silico* model of glucose metabolism was developed for the quantification of glucose uptake based on experimental data obtained from the MOC. The organs were allometrically scaled with respect to each other, and the flow rates were calculated based on physiological residence time of blood in each organ chamber [69]. The usage of automated robotic systems to study metabolism in multiple organ models has recently been proposed by Novak *et al*. [50]. The robotic interrogator maintained the viability and organ-specific functions of eight vascularized, two-channel organ chips (intestine, liver, kidney, heart, lung, skin, blood–brain barrier and brain) for three weeks in culture. The robotic interrogator and a physiological multi-compartmental model of the experimental system was used to quantitatively predict the glucose metabolism and distribution of an inulin tracer, a complex sugar, perfused through the platform [50]. Bauer *et al.* [70] presented a pancreas–liver MOC which allowed for the co-culture of human pancreatic islet microtissues and liver spheroids in an insulin-free medium. Similar to *in vivo,* hepatocytes take up glucose at a low level in the absence of insulin. Upon exposure to glucose, insulin was released by pancreatic islet microtissues, which stimulated an increased glucose uptake by liver spheroids. As the glucose concentration decreased, insulin secretion subsided, showing an efficient feedback loop between the liver and the insulin-secreting islet microtissues [70]. Glucose metabolism was also studied in another MOC integrating hepatocytes, adipose tissue and endothelial cells. This platform sustained glucose and fatty acid homeostasis *in vitro* over a period of 72 h. Afterwards, it was challenged with insulin and high glucose concentrations to mimic hyperglycaemia, and the ability to retain or restore physiological circulating glucose concentrations in response to insulin was investigated to determine the effects of these conditions on other metabolites involved in glucose and lipid metabolism. The authors demonstrated the impact of high glucose levels and their significant effects on the metabolic profile and insulin response [68]. In a follow-up study by the same group, two different scaling models were implemented in a hepatocyte–endothelial model—one based on cell numbers and the other based on metabolic rates and cell surface areas. An analysis of the metabolic response of the two configurations showed varying glucose and lipid balance, with the cell number-based scaling model displaying higher glucose consumption per hepatocyte and higher functional activity per cell than the metabolic rate-based scaling model [75].

In another system reflecting gut–liver–vascular interaction, Duan *et al.* [71] tested the hypothesis of fine particulate matter (PM2.5) influencing lipid metabolism. The main forms of exposure that were tested included gut to liver and liver to gut, highlighting oral and systemic delivery, respectively. In both cases, the PM2.5 particles were able to infiltrate the liver and gut cells and contribute to dysregulation of the cholesterol pathway in the liver first, followed by a dysregulation of the bile acid metabolism in the liver and gut [71].

Carbohydrate and protein metabolism was captured in a study by Maass *et al.* [72], who monitored gut, liver and cardiac OoCs over a period of one week in order to gauge nutrient consumption and metabolic profiles. Starting with the long-term assessment of the gut microphysiological system (MPS), the study was extended to a computational model of the gut, liver and heart MOC, highlighting the importance of understanding the metabolic needs of the individual OoCs not just from the perspective of carbohydrate metabolism but also from the perspective of metabolism of other species such as proteins [72].

For many years now, increased lactate levels have been consistently associated with morbidity and mortality in a wide range of metabolic disease states. To measure lactate consumption, a co-culture of human artificial liver microtissues and human neurospheres were exposed to fluid flow over two weeks in a multi-organ platform. Daily monitoring of lactate dehydrogenase (LDH) activity measurements in the medium and immunofluorescence endpoint staining showed the viability of the tissues and the preservation of differentiated cell phenotypes. Moreover, the lactate production and glucose consumption values of the tissues cultured indicated that a stable steady state was achieved after 6 days of co-cultivation. Toxicity testing was performed with the exposure of the system to the neurotoxin 2,5-hexanedione, which showed that the MOC responded with increased LDH release compared with the individual tissue cultures [73].

In a publication by Viravaidya & Shuler [74], a micro-scale cell culture analogue device was used to study drug metabolism. Their four-organ MOC captured physiologically based PK in a rat model on a microfluidic chip, highlighting drug toxicity, bioaccumulation and distribution [74].

## 4. Applications of metabolism-focused multi-organ chips

The previous chapters covered the fundamentals related to setting up MOCs to study a specific type of metabolism, along with some examples of state-of-the-art MOCs used to study metabolism. This section highlights case studies where the MOCs were applied towards two main areas—metabolic disease modelling and ADME/PD.

### 4.1. Metabolic liver disease modelling

Metabolic diseases represent a spectrum of disorders ranging from obesity, coronary artery disease, cardiovascular diseases, non-alcoholic fatty liver disease (NAFLD) and type 2 diabetes. Owing to an ageing population and the rise of obesity, this disease spectrum belongs to one of the fastest growing epidemics globally [76]. The pathophysiology of metabolic diseases is multifactorial and complex, with an increased incidence in industrialized countries because of the numerous environmental and genetic factors. NAFLD represents the most common chronic hepatic disorder in which fat is deposited in the hepatocytes. Metabolic injury to hepatocytes can lead to non-alcoholic steatohepatitis (NASH), which can result in liver fibrosis and cirrhosis. Although recent findings identified liver inflammation and toxic accumulation of lipids as the main molecular drivers for disease progression, it is not clear whether NASH develops sequentially based on fatty liver formation or whether it is a *de novo* event in a lipotoxic environment [77].

Developing new approaches to prevent or treat these diseases requires an understanding of the molecular mechanisms and signalling pathways that contribute to the disease. Although great progress has been made through genome-wide association studies in the identification of genetic variants or loci which play a part in metabolic diseases, the challenge remains to unravel the molecular mechanisms through which these genes contribute to pathophysiology. Animal models have been used to recapitulate metabolic diseases, but interspecies differences make it difficult to fully mimic human pathophysiology in clinical findings [78]. Furthermore, high failure rates have been observed, when efficacious concepts from pre-clinical testing were tested in clinical trials in patients, in which most of these drugs failed to provide convincing data regarding efficacy [79].

Here, we highlight what has been learnt from different models in which multi-tissue interaction has been shown to impact metabolic disease phenotype, with a focus on NAFLD and diabetes using MOC platforms.

#### 4.1.1. Multi-organ chips towards modelling non-alcoholic fatty liver disease

In a recent study by Yang *et al.* [80], a gut–liver-on-a-chip platform was created to study the initiation and progression of NAFLD. Microscopic high-content analysis and mRNA sequencing was combined to study the cross-talk between the gut and liver in the context of NAFLD. More so, NAFLD-induced FFA build-up was observed in the liver cells, alongside upregulated gene expressions linked with retinol metabolism and glucuronidation. Furthermore, accumulation of intracellular lipid droplets in the FFA-treated liver cells and gene expression patterns in relation to cellular endoplasmic reticulum stress was observed [80].

FFA uptake in the liver with subsequent lipid deposition in a liver model was also studied by Lee & Sung [81] to investigate processes of lipid absorption and induction of hepatic steatosis. In this model, anti-steatotic compounds mediated an improvement in a dysfunctional gut barrier and were able to ameliorate hepatic steatosis. By contrast, tumour necrosis factor α (TNF-α) treatment induced the progression of hepatic steatosis owing to compromised gut barrier function [81].

Further, Hughes and colleagues [82] established a combined gut–liver model to mimic NAFLD. In this study, the gut cultures were susceptible to inflammatory stimuli that decreased gut integrity, a similar process known to occur *in vivo* during NASH [82].

Although these *in vitro* NASH models served as ideal tools to understand the basic mechanisms of human NASH, none of these models served as a useful tool for assessing the efficacy of novel anti-NASH therapeutic compounds against a wide range of targeted pathways. The utilization of these micro-physiological three-dimensional steatosis liver platforms in an MOC would enable the study of cross-talk with organs involved in the development of NAFLD and provide valuable insights into glucose homeostasis in a physiologically relevant scale. Other notable models proposed include the liver–pancreatic islet communication by InSphero AG to study NASH.

A major limitation of the above models is that several NAFLD-associated organs such as the pancreas, white adipose tissue and kidney are neglected. To address this, previously established OoC modules integrating adipose [83], pancreatic [84], kidney tissue [85] and hepatic tissue [86] could be linked together. Thus far, organ cross-talk via bacterial metabolites, exosomes, miRNA etc. has not been mimicked. In order to study the impact of immune cell recruitment for disease progression, there is an added requirement of blood flow, cell sources from genetically diverse donors (e.g. PNPLA3, TM6SF2, MBOAT7 etc.) and bile acid recirculation (and modification by gut microbiota). Fibrosis as the main clinically relevant endpoint develops *in vivo* over years or decades and recapitulating this phenotype faithfully *in vitro* remains challenging. More scope for future study also relates to changes in bio-mechanics due to fibrosis and in identifying novel biomarkers to indicate the progression of NAFLD.

#### 4.1.2. Multi-organ chips towards modelling diabetes

Type 2 diabetes mellitus (T2D) is often linked with a high incidence of hepatic comorbidities, e.g. NAFLD. It is still debatable whether NAFLD is a consequence or a cause of pancreatic disorders [87]. Understanding the mechanisms which lead to T2D is important in the search for novel molecular drug targets to prevent and control this disease. Although the development of pancreatic chip models to study diabetes is still in the early stages, different pancreas-on-chip platforms are incorporated as co-cultures with other cell types [70,88]. A comprehensive overview of existing OoC models to study T2D research has been given by Rogal *et al.* [33].

Recently, a thermoplastic microfluidic-based pancreatic islet which automates islet loading and insulin sensing was used to deliver glucose pulses to positioned islets. Beyond glucose monitoring, the islets responded to other functionally relevant stimuli such as glucagon and amino acids. A perfusion with the suspension of human cadaveric islets confirms the capture of pancreatic islets in an automated manner with precise fluid control [65].

Zbinden *et al*. [84] recently developed an endocrine pancreas-on-chip model based on a tailored microfluidic approach, which allowed for self-guided entrapping of single human pseudo-islets. Human pseudo-islets were derived from the immortalized EndoC-βH3 cell line. This platform enabled precise control of vasculature-like perfusion, enabling prompt delivery of nutrients to the pseudo-islets and the excretion of metabolites. A unique addition is the incorporation of Raman spectroscopy to monitor the functionality of human pseudo-islets [84].

In another study, human pancreatic islet organoids generated from hiPSCs were perfused in a microfluidic platform. The islet organoids showed a similar tissue morphology and multi-tissue complexity mimicking human pancreatic islets *in vivo*. Moreover, the derived pancreatic organoids showed improved expression and maturation of β-cell-associated genes, insulin secretion and influx of calcium ions in response to glucose under dynamic culture conditions. This highlighted the role of biomimetic biophysical cues improving the islet organoid function and maturation, therefore serving as a promising tool for modelling diabetes *in vitro* and testing drugs for T2D therapy [89].

Substantial progress has been made in the development of multi-organ platforms emulating diabetes *in vitro*. Nguyen *et al.* [90] established an endocrine-on-chip system to model diabetes and to screen drugs for the treatment of diabetes by measuring insulin release over time. In this model, pancreatic and intestinal cells were co-cultured to quantify the effect of glucose on the release of glucagon-like peptide in the intestinal cells and insulin secretion from the pancreatic cell. In the study, glucose concentrations in response to different stimuli and change in the profiles of diabetes-associated genes were investigated. Despite the prospects that this model holds for diabetic therapy, a major limitation of this platform is the absence of a hepatic component, since T2D is often linked with hepatic comorbidities. Future platforms should incorporate liver cells to gain a better understanding of the multi-organ interplay in the disease process [90].

Mechanisms of T2D were further recapitulated in liver spheroids co-cultured with pancreatic islet microtissues to study liver–pancreas cross-talk, based on the regulation of glucose and insulin.

To assess the functionality of this model, the feedback loop between the liver and the insulin-secreting islet microtissues was recapitulated *in vitro*. The model served as a useful tool in understanding the mechanisms and comorbidities of T2D-associated diseases, including β-cell failure, insulin resistance, steatohepatitis and liver cirrhosis. However, the incorporation of other cell types such as white adipose tissue or kidney would be more metabolically representative [70].

Recently, the Leclerc group developed a pancreas–liver chip using rat islets and hepatocytes. The characteristic functions of the hepatocyte–islet co-culture model were evaluated and compared with monoculture conditions of the individual cell type. To assess pancreatic activity, the hepatocytes improved the islet response to hormonal cues as there was an increase in the secretion of insulin and changes in the expression patterns of the genes involved in regulating insulin and glucagon balance [91].

### 4.2. Absorption, distribution, metabolism and excretion/pharmacodynamics

Mechanistic toxicology is the identification and understanding of the cellular, biochemical and molecular mechanisms by which chemicals or drugs exert toxic effects. Mechanistic data are required to demonstrate adverse outcomes in risk assessment or to measure the relative toxic potential among the different species. In drug development, mechanistic studies are the key for repurposing studies of molecules that have an adequate PK profile (e.g. thalidomide).

MOCs allow for new perspectives in mechanistic toxicology that in the past could only have been addressed by animal or clinical studies. These platforms allow for the collection of data on biotransformation and the effect on target organs. They also allow for the simulation of kinetics in coordination with *in silico* data [92,93]. The possibility to monitor the generation of bioactive metabolites and their effect and/or accumulation inoff-target organs within the same MOC allows for the generation of complex human-relevant information before any *in vivo* study is performed. MOCs allow for designing ADMET studies that support mechanistic toxicology approaches because it is possible to use common *in vitro* toxicity methods such as cytotoxicity, cell viability and apoptosis, gene expression of metabolic enzymes and transporters, and combine them with non-invasive and clinically used toxicity biomarkers and advanced live imaging techniques. Additionally, diverse administration or exposure routes can be mimicked and compared, which could support future decisions on the ideal exposure route for a specific drug.

In the drug discovery process, the emergence of unexpected toxicity is often a problem resulting from a poor understanding of different drug metabolites. To assess the relevance of MOC for drug metabolism, several models have been developed. Materne *et al.* [73] presented a liver–neurosphere model which remained stable when connected, over a period of two weeks. The connected system seemed to be more sensitive to the neurotoxic drug 2,5-hexanedione than individual OoCs [73]. In a system reported by Wagner *et al.* [94], human liver microtissues and skin biopsies were studied for drug metabolism and inter-tissue cross-talk for up to 14 days of co-culture with trogalitazone [94]. Maschmeyer *et al.* [95] reported a complex system, capturing the absorption aspect via small intestine and skin models, the metabolism aspect via the liver and excretion via a kidney-on-chip system. They showed stable culture for up to 28 days, with two streams of circulating media—one circulating nutrients across the four organs, and the other ensuring drainage of effluent from the kidney epithelial compartment [95].

In a human-on-a-chip platform comprising models of the brain, pancreas, liver, lung, heart, gut and endometrium, with a mixer channel for the systemic circulation of tolcapone, tolcapone metabolism was investigated by analysing the supernatants in the medium using mass spectrometry. In this study, 12 different metabolites were identified, three of which were novel. These metabolites showed that reduction, oxidation and conjugation reactions are significant routes of drug metabolism [96]. In another study, four tissues—liver, heart, muscle and neurons—were integrated as a functional unit. In this model, primary cells and hiPSC-derived cells were used in the system under perfusion for 14 days, after functional analysis of the readouts in the system, interchange of metabolites and signalling molecules were exhibited. Furthermore, heart rate, muscle contractility, neuro-electrophysiology and production of liver albumin and urea were quantified and assessed; this served as an accurate model for predicting metabolism in different human organs [62]. In a follow-up study, the effect of cyclophosphamide on hepatic metabolic function was assessed. In a liver–heart co-culture cyclophosphamide induced cardiotoxic effects only after metabolism. Interestingly, the toxicity in the heart could be significantly reduced for the initially cardiotoxic terfenadine through metabolization [97].

Furthermore, Lee-Montiel *et al.* [98] recently created a multi-organ system consisting of hiPSC-derived hepatocytes and cardiomyocytes to study the metabolic conversion of cisapride to non-arrhythmogenic norcisapride through cytochrome P450 enzyme. This cisapride metabolism led to arrhythmia in the cardiac model. The authors were able to show functional integration of these systems allowing drug–drug screening and testing for toxicity [98]. Rajan *et al.* [99] developed an integrated system to accommodate six tissue constructs including liver, cardiac, lung, endothelium, brain and testes organoids. The tissues were incubated for 14 days; they were able to show that the metabolism of ifosfamide in their liver organoid produced chloroacetaldehyde and induced neurotoxicity. The platform represents an expandable, multi-organoid body-on-a-chip system that can be used for flexible characterization of drug interactions *in vitro* in a modular approach combining different organoid models [99].

A multi-layered chip was also proposed by Li *et al*. [100] that included tissues recapitulating functions of the liver, tumour, breast, lung and gastric tract. The system was used to assess drug metabolism, drug efficacy and toxicity in different organ-specific cells in parallel. The biomimetic organs-on-a-chip model not only captured the primary and secondary metabolism of capecitabine in different organs, but also enhanced the characterization of drug metabolism in a dynamic manner and its bioactivity on different organs in a simple approach [100].

In all these models, basic aspects of *in vivo* cross-talk of metabolic drug activity, their efficacy, mode of action and potential toxicity of off-target effects, which is critical for therapeutic interventions, could be mimicked with two or three organs in an *in vitro* platform. Most of these studies emphasized the inclusion of the liver because of its crucial role in entero-hepatic circulation as a central metabolizing organ to adequately mimic drug metabolization.

Lin *et al.* [101] studied the metabolism and toxicity of ciclosporin A in a liver–kidney MOC for its chronic nephrotoxicity and its role as substrate and inhibitor of CYP3A4 and *p*-gp. A 14-day repeated-dose systemic administration of ciclosporin A in combination with rifampicin from day 6 onwards showed the modulation of the biotransformation by the second drug at the hepatic level reducing mediated chronic nephrotoxicity. In this study, the authors used several clinical biomarkers for liver toxicity demonstrating the potential of MOC platforms for translational medical research [101].

The use of MOCs in mechanistic toxicology still faces numerous challenges, related to the confidence in the models, the perfusion settings, the selection of the ‘universal medium’, the inclusion of key components (endothelization and immune component) and the combination with systems biology approaches.

A recent assessment of the adoption of advanced cell culture systems (like OoCs) for toxicology studies and regulatory purposes revealed that model adoption depends on a detailed assessment of the model performance [102]. Ishida [103] defined the minimal requirements for systems mimicking the liver, small intestine and kidney epithelium covering cell culture properties such as transporter activity, membrane permeability and the capacity to withstand long-term culture. Cong *et al.* [104] reviewed the toxicity biomarkers already used in established single OoC systems covering hepatotoxicity, nephrotoxicity, cardiotoxicity and neurotoxicity. Indeed, the combination of physiological requirements and the use of translatable endpoints are essential for the application of MOCs in biomedical research, drug development and chemical toxicity [104].

A key consideration in the use of MOCs for ADMET studies is the perfusion system , which can be arranged as single pass or recirculating (reviewed in detail by Ronaldson-Bouchard *et al*. [105]). The use of complementary platforms that allow for both perfusion settings and the flexibility to test several directions of circulation would be beneficial to better answer questions related to bioactivation and positive or negative feedback responses. Also, the recapitulation of endothelial barrier functionality remains a challenge that should be addressed in future studies because of its relevance for systemic distribution and biotransformation of drugs in the ‘first-pass’ organ, the liver. Several single-organ chip models include an endothelial component but very few MOC models do. An initial approach of endothelization in a two-organ (liver and skin) model in 2015 enabled the evaluation of oral exposure with troglitazone to liver–intestine co-cultures as well systemic liver–skin co-cultures [106]. There exist other instances of ADMET studies being performed in MOCs [70,95,101,107–110]; however, no ADMET study has been performed so far with the presence of the endothelial component. More recently, Ingber and colleagues described a quantitative PK study in a vascularized organ-chip, characterizing the data from a first-pass MOC model perfusing a universal blood substitute between endothelium-lined vascular channels [50,111]: an arteriovenous reservoir enabled mimicry of drug distribution and dilution through the entire vasculature. The data compared closely with clinical data of cisplatin PK. This reinforces the importance of the commonly overlooked endothelial component contributing towards mitigating the challenge of a universal medium.

Most of the MOC publications addressing toxicological questions are focused on drug development [97,104,112]. Indeed, these cell culture platforms can provide valuable information in regard to chemical risk assessments here, but a significant need exists for replacing animal tests in (i) skin sensitization; (ii) repeated-dose toxicity; (iii) carcinogenicity; (iv) reproductive toxicity; and (v) toxicokinetics and quantitative *in vitro–in vivo* extrapolation (qIVIVE). Replacing these five systemic toxicity testing schemes requires a deep understanding of the possible mechanism of toxicity and the animal experiment cannot be replaced by an *in vitro* or *in silico* method on a one-to-one basis. As recently discussed by Veening-Griffioen [113], 'Tradition, not science, is the basis of animal model selection in translational and applied research'*.* MOCs and especially those combined with *in silico* modelling offer the possibility for a transition from current to future toxicology in safety science [114]. Also, the recent report from an EPAA Blue Sky Workshop suggested a workflow that allows for identification of suitable analogues for a target chemical and integration of toxicokinetic data to establish an acceptable level of exposure, or the incorporation of *in vitro* data to support the decision [115].

The contribution of MOCs for skin sensitization could be substantial. The general mechanisms of skin sensitization are well defined. A two-organ model with an immunocompetent skin chip [116] connected to a lymph node chip [117–119] allowing for topical exposure and for the perfusion of immune cells would suffice to quantify the four key events and adverse outcomes of the skin sensitization adverse outcome pathway (AOP) [120]. Carcinogenicity, especially of non-genotoxic chemicals, could be screened on MOCs combining the key administration and metabolism routes (e.g. gut, liver, skin and lung) connected to other key tissues when carcinogenesis is more likely to happen (e.g. pancreas, brain and kidney) and addressing the issues of metabolic activation of xenobiotics, causing immunosuppression or changes in cell death and proliferation at the tissue level [121]. The potential of using MOCs for repeated-dose toxicity comes hand in hand with carcinogenicity (recently reviewed by Yang *et al*. [122]). MOCs can be cultured under perfusion for many weeks and allow different combinations of organs that can be included to investigate their role on the toxicity and its molecular mechanisms. This is particularly important for *ab initio* studies of chemicals where other data and read-across methods do not provide sufficient information [115]. A current challenge in mechanistic toxicology is to bridge the data obtained from availability of advanced cell culture systems with systems biology (i.e. toxicogenomics) [123]. Addressing these challenges as well as the costs of these technologies will pave the way for merging MOCs with toxicogenomic approaches, high-content imaging as well as untargeted metabolomic approaches [124,125] covering cell products as well as chemical- or drug-related metabolites. These would place MOCs as essential tools for animal-free chemical risk assessment of human hazards.

Integrating *in silico* models with data from MOCs would create a powerful predictive tool for *in vivo* drug response or disease development. Table 1 summarizes the MOCs mentioned above. In the next section, we outline the framework to set up *in silico* models and potential applications for *in vitro–in vivo* translation.

**Table 1.** Summary of MOCs with applications in disease modelling and ADMET. GLP-1, glucagon-like peptide-1; GLUT, glucose transporter; hiPSC, human induced pluripotent stem cell; qPCR, quantitative polymerase chain reaction.

application	ref.	organ models involved	cell types	drugs studied	endpoints and assays performed
disease modelling (NAFLD)	[80]	gut, liver	Caco-2, HepG2	—	• microscopic high-content analysis • mRNA sequencing • gene expression
disease modelling (hepatic steatosis)	[81]	gut, liver	Caco-2, HepG2	turofexorate isopropyl (XL-335), metformin	• cell viability • permeability assay, • gut–liver chip-based drug screening
disease modelling (diabetes)	[65]	pancreas	human cadaveric islet and SC-β cell	—	hydrodynamic islet trapping insulin quantification
	[91]	pancreas–liver	primary rat islets and hepatocytes	—	RTqPCR assays
	[90]	intestine–pancreas	rat β-cell line INS-1, GLUTag cell line	GLP-1 analogues and natural insulin and GLP-1	ELISA immunoflourescence staining
ADMET	[73]	liver, brain	human liver spheroids and neurospheres	2,5-hexanedione	qRT-PCR toxicity assay endpoint tissue culture analysis
	[94]	liver, skin	HepaRG cell line, skin biopsies	troglitazone	multi-tissue sensitivity assay LDH assay
	[95]	human intestine, liver, skin, kidney	primary human intestinal constructs, HepaRG, skin biopsy construct, human proximal tubule cell line	selected drug candidates.	RNA quantification systemic tissue viability and barrier organ integrity
	[96]	brain, pancreas, liver, lung, heart, gut, endometrium	tissue constructs	tolcapone	metabolite profiling metabolomics
	[97]	liver, heart	iPS-derived cardiomyocytes, primary hepatocytes	cyclophosphamide, terfenadine	biomarker and enzyme expression electrical activity measurements drug quantification via HPLC-MS
	[98]	liver, heart	hiPSCs	cisapride, norcisapride, ketoconazole	drug transport studies, qPCR drug metabolism assays
	[99]	liver, heart, lung, endothelium, brain, testes	primary human cells, hiPSCs, cell line	capecitabine	drug toxicity assays
	[100]	liver, breast, lung, intestine	primary cells and cell lines	capecitabine	PK-PD studies, toxicity, and metabolism studies
	[101]	liver, kidney	cell line	ciclosporin, rifampicin	biomarker analysis protein expression
	[107]	testes, liver	primary human testicular organoids, HepaRG cells	cyclophosphamide	immunofluorescence hormone measurements
	[109]	liver, skin	human epidermal cells, HepaRG	hyperforin, permethrin	gene expression qPCR mass spectrometry
	[111]	intestine, liver, kidney; bone marrow, liver, kidney	Caco-2, primary human hepatocytes, human renal proximal tubule cells, primary bone marrow progenitor cells	nicotine, cisplatin	barrier function assessment mass sprectrometry flow cytometry

## 5. *In silico* modelling of multi-organ chips and *in vitro*–*in vivo* translation

Pre-clinical and clinical drug development calls for the investigation of PK-PD parameters of a drug candidate by testing with a variety of *in vitro* and *in vivo* models. Experimental and computational models could complement each other in the prediction of drug–body interactions. Computational models could also enable the prediction of potential metabolic pathway disruptions, leading to diseases.

MOCs are now being used to define PK parameters for various drugs [32,111]. They allow for the study of the fate of a drug when exposed to a functional organ tissue, to determine PK properties such as absorption, distribution, metabolism and excretion. This opportunity comes from the advantage of the ability to monitor effluents and to study response parameters in real time with the help of sensors or downstream effluent analysis and hence complete control over the characterization of a model and the parameters that go with it. This has led to the investigation of multi-compartment bioreactor models to demonstrate pharmacological interactions between organ models [43]. In order to use these pharmacological data to be able to predict the dose requirements in a clinical setting, there arises a need to create integrated models which require more inputs than flow rate and PK parameters. These inputs include metabolites, externally administered and secreted soluble factors, and a model shift from PK-PD to a more physiologically based PK (PBPK)-PD model where the outputs consist of response markers such as cytokines and growth factors which could then influence the progression of the drug response. Yu *et al.* [126] demonstrated this with an example of a traditional PK model and a mechanistic model for concentrations of hydrocortisone (HC), which when not bound to human serum albumin (HSA) can be metabolized by the liver. The mechanistic PK model accounted for HC-HSA binding and produced a constant value of *k*_metabolism_ for different values of initial bound/unbound HC ratio, which could be used to study drug PK *in vivo,* compared with the traditional PK model, which did not consider the mechanism of HSA binding [65]. Such studies lay the foundation work for *in vitro* to *in vivo* extrapolation (IVIVE), where MOCs could be used to predict *in vivo* PK properties of the drug [127].

So far, *in silico* models have evolved along a spectrum in terms of the amount of detail that they capture. A mini-review on these models by Helmlinger *et al.* [128] displays the evolution of *in silico* modelling approaches depending upon the application. There exist models that focus solely on intracellular mechanisms, and these follow the principles of systems biology. At the other end of the spectrum there exist computational models which represent a human as a collection of organ ‘compartments’ and these are the PBPK models, where the functions of the compartments are quantified on a large scale, and this general interaction can predict PD of the drug when interacting with this system [129]. Quantitative system pharmacology (QSP) models allow for intracellular detail depending upon the scientific question and can still maintain the broad interaction between different organs within the model undergoing said interaction. The European Union Reference Laboratory for alternatives to animal testing (EURL ECVAM) is heavily involved in understanding the current state of affairs of *in silico* modelling and requirements to bridge the gaps in regulatory acceptance of *in silico* models [114,129–132].

### 5.1. Framework of *in silico* modelling in multi-organ chips

**Figure 3.**
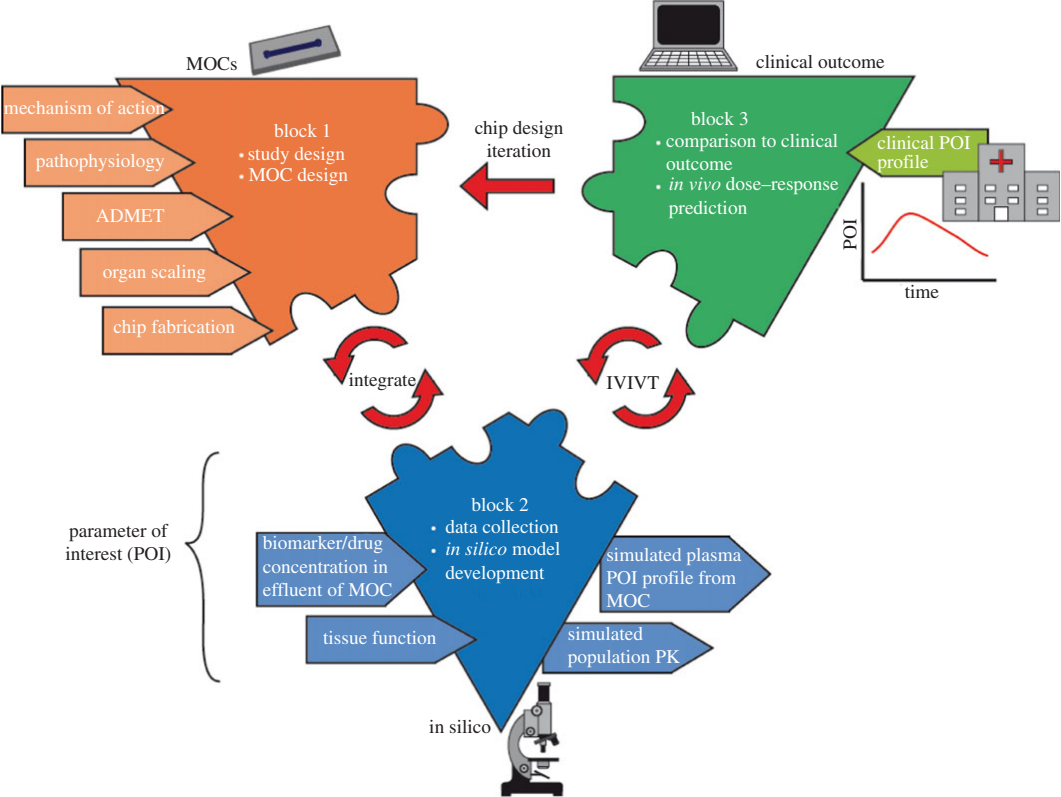
Framework for the integration of MOCs and computational model data to predict *in vivo* outcomes.

The process of correlating MOC data and *in silico* models with *in vivo* responses consists of three blocks (figure 3).

#### 5.1.1. Block 1—study decisions and multi-organ chip development

In block 1, the MOC is developed, based on the type of study and organs involved. The mechanism of action of the drug or disease pathophysiology is determined, in addition to the decisions about the involvement of the immune component.

To use MOCs for PBPK-PD studies, the organ models should be designed and appropriately scaled to maintain a physiological relation similar to the *in vivo* situation. A compilation of scaling approaches has been provided by Ahluwalia [133]. Simple allometric scaling relies on using the quarter power law to scale weight/volume of organs down from macro-scale to micro-scale in correlation with the whole-body weight [134]. Wikswo *et al.* demonstrated that this approach leads to disproportionately sized micro-organs with respect to each other and fails to capture how organ functions scale with respect to each other [39]. Shuler and colleagues did some fundamental work on organ model scaling considering specific organ mass and the residence time of blood within the organs. Briefly, each organ was scaled according to organ mass or volume and the blood flow into each chamber based on *in vivo* residence times, then ordinary differential equations were used to calculate time-specific concentrations per compartment of drugs and metabolites [42,66,74]. For unknown mechanisms of toxicity, it is crucial to scale tissues with respect to their functionality. The third method of scaling accounts for the functional property of the organ and aims to scale that to the micro-scale, while additionally considering the spatial arrangement of cells in an MPS platform. An important aspect of this approach involves accounting for the distribution of resources to the organ tissues *in vitro* compared with *in vivo. In vitro*, the cells are in a resource-rich environment, and this would cause them to behave differently from their *in vivo* counterparts. Moraes *et al.* [135] investigated insulin-dependent glucose uptake in adipose tissue and discussed how the spatial arrangement of cells could influence the functional activity, for example the metabolic activity. They also highlighted considerations for scaling organs based on volume or surface area (three- or two-dimensional) depending on whether the organ is functionally three-dimensional (secretory, storage functions of glands, bone marrow and fat) or functionally two-dimensional (filtration, absorption, molecular transport functions for membrane organs such as endothelial cells, kidney, the blood–brain barrier and lungs) [135]. Other work on functional scaling has been done by West *et al.* [136] and Toussiant *et al*. [137]. The organ scaling process is iterative and requires the use of computational mechanistic models to validate the quantitative data obtained from the model and, in the process, helps to refine the scaling parameters to iterate the model [136,137]. Maass *et al.* [72] show that mechanistic computational models enable an improved design of MOCs by highlighting the medium component requirements of each connected organ at a steady state to enable longer term pharmacological studies. In a novel computational multi-organ scaling approach, the functional scaling in comparison with allometric and direct scaling approaches was investigated. In the study, an objective function was defined that represents the biological functions of interest and aims to minimize the discrepancy between model-derived and previously established data [138].

To expand the effectiveness of the IVIVE study, it is important to consider the following points about the system: age, sex and body mass index could change the extent of organ function—a number of studies demonstrate that plasma concentrations of glucocorticoids and progestins are significantly lower for obese women than for women of normal weight. Immune responses–drug treatment could trigger the immune system and the pathways for these processes have not been well defined. Many drugs that were not intended for immune modulation could trigger immune side effects [139]. Traditional PK models can capture their effect only to a limited extent, while PBPK-PD models or mechanistic PK models could be coupled with multi-organ MOCs to predict the impact of the drug-related immune response on the metabolism. Trapecar *et al.* [140] show this as well in a multi-organ ulcerative colitis model including liver, gut and circulating T_reg_ and Th_17_ cells. They note that short-chain fatty acids incite an immune response from CD4^+^ cells, which in turn leads to a disrupted gut barrier and reduced liver function [140].

#### 5.1.2. Block 2—gathering multi-organ chip data and *in silico* model design

Experimental data derived from MOCs under highly reproducible conditions providesa valuable basis for mechanistic *in silico* modelling of the observed metabolic effects. Essential parameters such as glucose turnover and oxygen consumption could be continuously monitored by integrated glucose and oxygen sensors and its change linked to treatments with different drug dosages. These data could be complemented by specific biomarkers, cytokine profiles and drug metabolite formation. Maass *et al.* [72] studied the effects of medium renewal on tissue function and validated their quasi-steady-state model by performing experiments to observe the effect of medium change on tissue function. These data were correlated to observed and predicted concentrations of medium components such as glucose and lactate. For their study, the glucose and lactate concentrations were kept within physiological ranges (a glucose concentration of 4–7 mM and lactate concentration below 20 mM). They reported that 15% of medium change every 24 h is enough to maintain functional properties of the tissue cultures in their model (iPS-derived cardiomyocytes, primary human hepatocytes and differentiated Caco2 intestinal cell line), allowing for *in silico* models to predict strategies for the judicious use of medium components within a physiological range [72].

General considerations to be made while running metabolic *in vitro* studies include: (i) the role of serum present at different levels in the medium and the impact of drug binding, i.e. by albumin [141–143], (ii) concentrations of medium components and how they relate to the *in vivo* situation, (iii) differences between cell sources and functional variations between cell lines or cell types, and (iv) the material used for MOCs and its drug absorption/adsorption characteristics.

In order to characterize the PK-PD properties of the drug in the MOC, it is essential to set up a baseline drug profile within the system in the absence of cells, in order to establish a blank ‘system control’. Partitioning of lipophilic compounds into PDMS could influence the initial drug concentration that the cells are exposed to. Rigid plastics mitigate this issue but have lower levels of oxygen permeability than PDMS.

#### 5.1.3. Block 3—*in vitro* to *in vivo* translation

The *in silico* model can be used to predict *in vivo* outcomes based on physiologically based PK modelling or systems pharmacology approaches and validated against the *in vivo* situation based on clinical biomarkers and drug serum levels. Criteria for the MPS validation are the time-dependent changes of drug levels in blood against unbound drug levels in the medium perfusate of the MOC.

Quantitative systems PK has been extended from an approach to determine PK for therapeutic development to understanding relations between MOCs from a biological and physical dynamics standpoint. This can then aid defining appropriate experimental conditions and predict *in vivo* outcomes. Edington *et al.* [93] applied the principles of quantitative systems pharmacology (QSP) to determine the conditions required to maintain functionality of up to 10 organ tissues connected on a flow platform with a built-in pumping system. The platform was used to measure metabolite concentrations in effluents and to calculate metabolite formation rates. The organ model platform was further used to quantify diclofenac metabolism and the correlation of the effluent measurements with the PBPK model [93].

Arakawa *et al.* [144] created an MOC model of Caco2 cells and HepaRG cells representing cross-talk between intestine and liver tissue. The obtained data were used to predict plasma profiles of triazolam in humans. The authors used scaling factors to predict blood plasma concentration of metabolites and triazolam in humans [144].

Tsamandorous *et al.* [145] performed an array of tests on cryopreserved hepatocytes from five different donors to assess drug effects and the possibility, by using a simulation framework, to predict *in vivo* drug metabolism. They assessed hepatocyte functionality through albumin secretion and expression of metabolism-related genes upon exposure to six different drugs and were able to successfully predict *in vivo* clearance of drugs [145].

A number of software tools can be employed to solve the equations generated to set up the *in silico* model. Ordinary differential equations are generated to describe the time-dependent variation of concentration of a component within the MOC system. Common software for ordinary differential equation modelling includes R, Matlab, SAAM2, Berkeley Madonna [146] and others. Examples of more integrated platforms for PBPK modelling are the OSP Suite from Bayer/esqLABS [147], SimCYP [148] from Certara and GastroPlus [149]. For QSP modelling, the OSP Suite from Bayer/esqLABS is more widely used.

The MOC field is rapidly growing, proven by advances in hardware technology to culture cells, perfuse media and integrate multiple organs in a single platform with advanced sensor arrays. Likewise, the recapitulation of human physiology *in vitro* is rapidly advancing. However, an integrated software solution to streamline MOC experiments within the drug development process is still lacking. A software solution may guide experimental designs, provide model-informed selection of drug concentrations to analyse *in vitro* data and translate it into clinical outcomes predictions by using human QSP/PBPK models. Individual reports have demonstrated the predictive power of such an approach over conventional methods already. Further adoption and development are now needed to fully demonstrate the impact of *in silico* and *in vitro* models on the drug development process with improved prediction of drug efficacy, a reduction of attrition rates and identification of as yet non-clinically detectable toxicity effects.

### 5.2. Case studies

While there are a considerable number of publications on MPSs, publications of studies that combine computational modelling and MPS-derived experimental data are scarce. A Pubmed query (pubmed.ncbi.nlm.nih.gov) including the search terms (OoC) or (MPS) resulted in 2733 entries from 2013 to 2021. When including search terms such as PK, PBPK and QSP, 103, 12 and 12 publications, respectively, were found (corresponds to 4%, 0.5% and 0.5% of the total entries). Thus, only a handful of reports directly report computational analysis of the biological data and the subsequent integration with human-based PK or PBPK/QSP models. In this section, we highlight two of these reports as example case studies.

#### 5.2.1. Assessing the toxicodynamic effects of terfenadine on a heart–liver multi-organ chip

In a publication by McAleer and co-workers, the authors proposed a workflow integrating both experimental data and computational models to inform pre-clinical outcomes after terfenadine administration causing QT-prolongation [150]. They investigated the potential of MOCs to recapitulate pre-clinical PK-PD relationships of terfenadine. The toxicodynamic effects of terfenadine on the heart, which are pre-clinically and clinically well characterized, are caused by QT prolongation, which was quantified by changes in the field potential duration in a heart MPS. It is also known that mainly CYP3A4 enzymes metabolize terfenadine to fexofenadine, which does not cause any QT prolongation. The authors then fluidically coupled the heart MPS to a liver MPS. The aim was to demonstrate that, in the presence of a metabolically competent MPS, the toxicodynamic effects on the heart MPS were less pronounced, as soon as terfenadine was metabolized to fexofenadine. Therefore, they quantified generic biomarkers such as cell viability and MPS-specific biomarkers such as the heart-MPS beat rate or the field potential duration over the course of the experiment (24 h). Additionally, they quantified both terfenadine and fexofenadine concentrations in the cell medium and lysate of the liver and heart MPSs (multiple time points over 24 h). The authors confirmed the presence of fexofenadine and thus indirectly confirmed that the liverMPS was metabolically competent and could indeed metabolize terfenadine to fexofenadine via CYP3A4. The authors then developed a four-compartmental mathematical model of the multi-MPS to describe the kinetics of both MPS-specific biomarkers and drug concentrations. They even accounted for the absorption of highly lipophilic compounds (such as terfenadine) to the multi-organ platform, which is mainly made of PDMS and would reduce the actual drug concentrations available for metabolism or adverse effects. After the mathematical model was calibrated to describe the heart–liver system biological data, the authors used this model to predict the change in FPD in animal models (guinea pig and dog) as well as humans. This was done by providing the *in vitro* model with terfenadine kinetics taken from available literature reports.

#### 5.2.2. Predicting clinical outcomes after administration of cisplatin in a kidney immune *in silico* model

In a recent publication by Maass *et al.* [151], a different workflow was applied by integrating both experimental data and computational models to inform clinical outcomes after cisplatin administration causing acute kidney injury (AKI). A PBPK model was developed to describe the kinetics of cisplatin in humans (based on literature reports). Especially for the kidneys where damage is observed *in vivo*, the model was used to inform the drug concentrations that should be tested in organ chips. The toxicodynamic effect of cisplatin was quantified by measuring cell viability and the kidney-specific biomarker KIM-1 (kidney-injury molecule). This biomarker is routinely used in clinical studies for the assessment of AKI. KIM-1 profiles were compared between a kidney chip model and two-dimensional cell culture. Following the *in vitro* experiments, the authors developed another PBPK model to describe the distribution and synthesis of KIM-1 in humans. This model served as an *in silico* MOC model. This model included an immune system response (neutrophil recruitment) causing elevated KIM-1 levels. The experimentally derived biological data on KIM-1 kinetics after cisplatin administration (multiple time points over the course of the experiment) as well as the simulated drug concentration kinetics were used as inputs into this newly developed human PBPK model. The authors successfully described the kinetics of KIM-1 in humans as a function of drug concentrations at the site of action, which was informed by the *in vitro* experiments. The results were compared with a list of clinical reports on elevated KIM-1 levels in patients with AKI . This demonstrates the power of integrating *in vitro* and *in silico* approaches.

These two exemplary studies demonstrate the power of combination of MOC experimental data with computational modelling to identify drug-related toxicity in pre-clinical studies to inform the dosing regimen and to predict clinical outcomes.

The ability to precisely and accurately predict adverse events and efficacy in pre-clinical studies could help to reduce patient burden and drug attrition rates. Yet current animal models and state-of-the-art *in vitro* systems fail to do so. The examples presented above show the potential of the translational workflow in two specific applications. Future studies may extend the approach for various translational pharmacology applications, such as toxicity assessment, first-in-human dosing and metabolism. The adoption and validation of the presented workflow would include testing a broader set of drugs, measure platform and medium binding of compounds routinely, and identify clinically relevant and organ-specific biomarkers. More studies highlighting the predictive power of an integrated approach over conventional methods (*in vitro–in vivo* correlation/extrapolation, two- versus three-dimensional or MPSs, animal testing) in the assessment of drug-related clinical toxicity and efficacy testing are needed.

## 6. Outlook

With advances in microfabrication techniques, three-dimensional cell culture methods, stem cell technologies and biomarkers, the development of OoC technology has been rapid over the last decade [25]. As a result, multiple ready-to-use OoC models are already commercially available, for biomedical research, toxicity screening and drug testing [152]. In a recent publication, the question of how much impact MPSs could have in early drug discovery (high-throughput screening) and pre-clinical studies was investigated and estimated to save up to 25% (approx. US$700M) of total research and development costs per drug candidate [153]. While both industry partners and regulatory agencies are starting to recognize the impact and predictive power that OoC systems may have on drug testing, the full potential of the technology is not used yet.

Mastrangeli *et al.* [154] highlighted six components in the development of OoC technology that the field is making headway in, in one form or another. However, certain limitations are to be overcome for OoCs to get approval by regulatory bodies. Table 2 provides a summary of the challenges faced by the MOC field. Allwardt *et al.* [155] also conducted a survey consolidating the challenges of the OoC field. Alongside a roadmap of requirements for the technology, they highlighted challenges and these are considered at Technology Readiness Level 4 (technology validated in the laboratory) [155].

**Table 2.** A summary of requirements of the MOCs and the current challenges that the field still faces. The reader is referred to specific sections within the manuscript for more details about each challenge.

challenge	important aspects	refer to section
connection strategies	• flexibility to allow modular connection of organ chips with different requirements/time lines for tissue formation and maturation • minimizing dead volumes • leak-proof and robust connection mechanisms	2
standardization	interfacing with automated sampling approaches and modular connection approaches connection of chips from different developers	2
physiologically relevant microanatomy	cell sourcing (human, autologous cell types) maintaining tissue complexity and homeostasis in individual organ modules in connected culture immune cell integration (resident and peripheral)	1, 4
chip material	biocompatibiliy tuneable gas permeability non-absorbing versus case-by-case small molecule absorption characterization transparency for imaging economical production for prototyping/pilot/large scale	2
time-resolved analyte quantification	limited number of analytes of in-line sensing strategies assay sensitivity and volume requirements of off-line assays	2, 3, 4
clinical translation	clinically relevant endpoints *in silico* models for IVIVT and dosing predictions	5

In order for OoCs to be taken up as a drug-screening platform, either an ‘evolutionary’ approach or ‘revolutionary’ approach is needed, as highlighted by Heringa *et al.* [156]. In the evolutionary approach, OoCs could be compared with animal data for toxicity endpoints, after which the validated OoC model could be used to derive safe limits for humans. The revolutionary approach seeks to redefine toxicity endpoints by the development of quantitative AOPs and to use OoC and *in silico* data to predict the drug response in humans. In both cases, *in silico* modelling could provide a bridge for *in vivo* prediction and strengthen the data obtained from the OoC.

To increase trust in the novel technological advances, collaborations of academics, industry partners and regulatory agencies should prove that OoCs are better predictors of human outcomes and establish best practices for using commercially available OoCs. There is an immediate need for an integrated workflow that combines both computational models and data derived from MPS experiments (i.e. *in vitro–in vivo* translation (IVIVT)). Such a translational workflow may enable the assessment of toxicity and safety hazards, could inform first-in-human drug dosing and may help to identify potential drug failures pre-clinically, reducing time, cost and attrition rates.

Diseases associated with metabolism tend to be chronic, i.e. NAFLD and NASH, which could proceed to hepatocellular carcinoma not only in adults but also in children and adolescents, especially with the spike in obesity [157]. The global prevalence of NAFLD is 24% and these chronic illnesses are associated with metabolic syndrome, which is characterized by increased visceral fat, insulin resistance and circulating fatty acids. Another important facet of OoC technology is the ability to integrate immune cells, which are involved in many hepatotoxicity responses to drugs.

Current therapeutic regimes are primarily designed for the average patient, based on the observed benefits and success rates within the general population, without considering the tremendous genetic diversity between individuals [158]. Such a uniform treatment strategy bears the risk of significant adverse events and the administration of inappropriate drug dosages with insufficient drug efficacy. Therefore, the clinical need for patient-specific treatments, based on individual genomic backgrounds, remains [159]. Microfluidic MOCs, using patient-derived cells, represent a promising tool to such personalized medicine. While the utility of mature differentiated cells is often limited owing to their low regeneration, the use of induced pluripotent stem cells (iPSCs) may overcome this drawback. Indeed, iPSCs show a remarkable capacity of cell renewal and a potential to differentiate into a plethora of cell types. However, it should be mentioned that such iPSC-based techniques are not yet up to the mark, causing a high level of heterogeneity, or immature phenotype, in the derived cell types [160,161]. Improved differentiation protocols are thus needed to generate more mature cell types with a higher homogeneity and lower batch-to-batch variation. Additionally, to allow for high-throughput screening of compounds, the parallelization of MoCs should be possible [162]. Once fully optimized, these personalized MoCs may provide a tool for long-term tests, mimicking the biological environment, to predict the response of multiple organs to a specific drug and to define appropriate dosage, therefore elevating the potential for a beneficial clinical outcome. For example, MoCs with integrated circulatory systems could have high potential in oncology, as they might predict the tissue to which metastatic cells will migrate [163].

The key values to obtain a high translational value of MOCs relies on the recreation of a biologically relevant on-chip environment, the use of suitable and sustainable cell models and the availability of sensitive and reliable readouts [164]. Indeed, while many of these features have been addressed by current research, significant improvements of the OoC models are still required to allow their integration into the drug development process and even diagnostics. A key asset in the adoption of OoC systems is the ability of *in silico* modelling to contribute to the predictability of the OoC system. Another aspect includes the cell source—while most OoC models still use cells from animal origin, more advanced models will integrate cells of human origin, i.e. primary cells or iPSC-derived cells. Current OoC models are not yet able to fully reflect the key characteristics of *in vivo* cellular homeostasis or pathology, such as zonation of tissues, zonal pressure, integration of a complex microbiome, physiological fluctuation of hormones or integration of a full-scale organ-specific immune response [105,164,165]. However, emerging techniques and materials, such as the use of novel biomaterials (considering the importance of cell–material communication), integration of high-throughput microfluidic systems (allowing parallel experimenting) and integration of novel sensor arrays will contribute to improving current OoC platforms [166]. We are thus confident that the significant advancements in MOC technology will enhance and encourage its broader use in biological and clinical research and help to transform non-clinical research towards precision medicine with improved capabilities of personalized disease modelling and drug discovery.

## Data Availability

This article has no additional data.
